# Diagnostik und Therapie der Lupusnephritis – 2023

**DOI:** 10.1007/s00508-023-02263-8

**Published:** 2023-09-20

**Authors:** Balazs Odler, Marion J. Pollheimer, Andreas Kronbichler, Marcus D. Säemann, Martin Windpessl, Philipp Gauckler, Michael Rudnicki, Emanuel Zitt, Irmgard Neumann, Karl Lhotta, Kathrin Eller

**Affiliations:** 1 Abteilung für Nephrologie, Innere Medizin, Medizinische Universität Graz, Graz, Österreich; 2 Institut für Pathologie, Medizinische Universität Graz, Graz, Österreich; 5 6. Medizinische Abteilung mit Nephrologie & Dialyse, Klinik Ottakring, Wien, Österreich; 6 Abteilung für Innere Medizin IV, Klinikum Wels-Grieskirchen, Wels, Österreich; 7 Medizinische Fakultät, JKU, Linz, Österreich; 8 Department Innere Medizin 4 (Nephrologie und Hypertensiologie), Medizinische Universität Innsbruck, Innsbruck, Österreich; 9 Abteilung für Innere Medizin III (Nephrologie, Dialyse und Hypertensiologie), Akademisches Lehrkrankenhaus Feldkirch, Feldkirch, Österreich; 10 Vasculitis.at, Wien, Österreich; 11 Immunologiezentrum Zürich (IZZ), Zürich, Schweiz; 12 Medizinische Fakultät, SFU, Wien, Österreich

**Keywords:** Immunsuppression, Lupusnephritis-Stadien, Proteinurie, Supportive Therapie, Immunosuppression, Proliferative lupus nephritis, Proteinuria, Supportive therapy

## Abstract

Das vorliegende Manuskript fasst die Empfehlungen der Österreichischen Gesellschaft für Nephrologie zur Diagnose und Therapie der Lupusnephritis zusammen und erläutert die Hintergründe der entsprechenden Empfehlungen anhand der vorhandenen Literatur. Wir besprechen im Detail die immunsuppressive Therapie in proliferativen Stadien der Lupusnephritis (Stadium III und IV mit/ohne Stadium V) und in der Lupusnephritis im reinen Stadium V mit großer Proteinurie. Zudem wird auch die konservative, supportive Therapie der Lupusnephritis detailliert besprochen. In den Abbildungen haben wir versucht, einen Leitfaden für die Praxis zur Therapie der Lupusnephritis zu erstellen.

## Abstract (en)

The manuscript summarizes the consensus of the Austrian Society of Nephrology on the diagnosis and therapy of lupusnephritis, which is built on existing studies and literature. We discuss in detail the immunosuppressive treatment in proliferative forms of lupusnephritis (III and IV ± V) and in pure lupusnephritis V with nephrotic-range proteinuria. Furthermore, the supportive medication in lupusnephritis is summarized in the consensus. The figures were designed to provide the reader a guidance through the therapeutical approach in lupusnephritis for the daily practice.

## Einleitung

Der systemische Lupus erythematodes (SLE) ist eine chronische Autoimmunerkrankung, die vorwiegend Frauen im gebärfähigen Alter betrifft. Interessanterweise weisen Männer mit SLE häufig einen schwereren Erkrankungsverlauf auf. Die meisten Patient*innen mit SLE entwickeln mehrheitlich im frühen Krankheitsverlauf eine Nierenbeteiligung während ihrer SLE-Erkrankung. Eine klinisch relevante Nierenerkrankung tritt bei mehr als 50 % der Patient*innen mit SLE auf, und etwa 10 % der Patient*innen mit Lupusnephritis (LN) entwickeln in Folge eine chronische Nierenerkrankung (CKD) bis zum Stadium 5. Patient*innen, die aufgrund einer LN eine CKD im entwickeln, haben ein 3‑fach erhöhtes Sterberisiko [1, 2]. Die Inzidenz der LN bei Patient*innen mit SLE bist aufgrund der Ethnizität sehr unterschiedlich, da die Inzidenz von LN bei Patient*innen mit schwarzer, hispanischer und asiatischer Herkunft deutlich höher als bei weißen Patient*innen ist [2].

## Klinik und Diagnostik

Der SLE wird nach den Kriterien der European League Against Rheumatism/American College of Rheumatology (EULAR/ACR) aus dem Jahr 2019 diagnostiziert [3]. Das Vorliegen einer LN sollte bei Patient*innen mit bekanntem oder vermutetem SLE erwogen werden, die ein aktives Harnsediment und/oder Proteinurie oder Albuminurie entwickeln. Letztere werden mittels Urin-Protein/Kreatinin Ratio (UPCR) oder Albumin/Kreatinin Ratio (UACR) im Spontanharn bestimmt. Erhöhte Antikörper gegen doppelsträngige DNA (Anti-dsDNA) und niedriges Komplement (C3 und/oder C4) sowie eine hohe Erythrozytensenkungsrate können auf einen aktiven SLE mit LN hinweisen. Wir empfehlen außerdem, Patient*innen mit bekanntem SLE routinemäßig durch Harnsedimentanalyse sowie UPCR und UACR zu untersuchen. Bei Patient*innen mit Verdacht auf LN (persistierende abnorme Proteinurie – UPCR > 0,5 g/g mit oder ohne aktivem Harnsediment (dysmorphe Erythrozyten (≥ 5 Zellen) und/oder Zylindern) und/oder eine verringerte oder abnehmende glomeruläre Filtrationsrate (GFR) ohne zurechenbare Ursache) sollte eine Nierenbiopsie durchgeführt werden, um die LN zu diagnostizieren und in Folge den histologischen Subtyp der LN und die Aktivität/Chronizität der LN zu bestimmen. Wie bei jedem invasiven Verfahren ist jedoch eine individuelle Nutzen-Risiko-Abwägung erforderlich. Eine erneute Nierenbiopsie sollte bei Verdacht auf ein LN-Rezidiv durchgeführt werden, das sich durch eine Zunahme der Proteinurie bzw. Albuminurie, ein aktives Harnsediment und/oder einen Anstieg des Serumkreatinins zeigt. Darüber hinaus kann bei Patient*innen in klinischer Remission auch ein Ausschleichen oder ein Absetzen der Immunsuppression mittels Nierenbiopsie indiziert werden [2].

## Histopathologische Beurteilung

Die Nierenbiopsie mit Bewertung der histopathologischen Klasse ist nach wie vor der Goldstandard für die Diagnosestellung, die Bewertung der Krankheitsschwere und die Festlegung geeigneter therapeutischer Strategien bei LN [4]. Für eine zuverlässige histopathologische Diagnose und Klassifizierung der LN sind eine angemessene Gewebeprobe, die mindestens 10 Glomeruli für lichtmikroskopische Analysen enthält, und geeignete histopathologische Techniken, unter anderem eine optimale Konservierung und Verarbeitung, unerlässlich [5, 6]. Histologisch ist die LN häufig durch eine erhebliche Heterogenität gekennzeichnet, die Glomeruli, peritubuläre Kapillaren, muskuläre Blutgefäße und das Interstitium betrifft. Eine Nierenpathologie ist oft durch eine mesangiale Hyperzellularität, eine verdickte glomeruläre Basalmembran, eine endokapilläre Hyperzellularität, glomeruläre Nekrose, Halbmondbildung, Anzeichen einer thrombotischen Mikroangiopathie sowie tubulointerstitielle Entzündung gekennzeichnet. Für eine vollständige histopathologische Abklärung ist die Durchführung spezieller Färbungen und Immunfluoreszenzanalysen für IgG-, IgA- und IgM-Isotypen, Kappa- und Lambda-Leichtketten sowie die Komplementkomponenten C3 und C1q nötig [6]. Glomeruläre Immunablagerungen enthalten dominantes polyklonales IgG, das als wesentlich für die Diagnose angesehen wird, in der Regel begleitet von IgM-, IgA-, C1q- und C3-Koablagerungen (Full House) [6]. Die Elektronenmikroskopie (EM) zeigt in der Regel elektronendichte Immunablagerungen in allen glomerulären Kompartimenten (Mesangium, subendothelial und subepithelial) und weist typischerweise ein Daumenabdruckmuster der Immunablagerungen auf [6, 7].

Gemäß der Klassifikation der *International Society of Nephrology* (ISN)/*Renal Pathology Society* (RPS) von 2003 weist eine LN der Klasse I lichtmikroskopisch normale Glomeruli, aber mittels Immunfluoreszenzanalyse nachweisbare mesangiale Immunkomplexablagerungen auf [4, 6]. Die LN der Klasse II zeigt eine mesangiale Hyperzellularität (definiert als drei oder mehr Mesangialzellen pro Mesangialfläche in einem 3 Mikrometer dicken Schnitt) in Verbindung mit mesangialen Immunablagerungen. In den Klassen III (fokal) und IV (diffus) werden glomeruläre Narben und/oder proliferative, nekrotisierende und sichelförmige Läsionen festgestellt, die weniger als 50 % (III) oder mehr als 50 % (IV) der Glomeruli betreffen [4, 6]. Diese Läsionen werden als aktiv (A), chronisch (C) oder beides (A/C) eingestuft. Aktive Läsionen umfassen das Vorhandensein von endokapillärer Hyperzellularität mit oder ohne Leukozyteninfiltration und mit erheblicher Luminalverkleinerung, Karyorrhexis, fibrinoider Nekrose, Ruptur der glomerulären Basalmembran, Halbmonden, zellulären oder fibrozellulären, subendothelialen Ablagerungen, die durch Lichtmikroskopie identifizierbar sind (Drahtschlingen), und intraluminalen Immunaggregaten (hyalinen Thromben). Zu den chronischen Läsionen gehören segmentale oder globale glomeruläre Sklerose, fibröse Adhäsionen und fibröse Halbmonde [6].

Im Jahr 2018 hat eine internationale Arbeitsgruppe führender Nephropathologen Aktualisierungen des ISN/RPS-Klassifizierungssystems vorgeschlagen (siehe Tab. 1), um problematische Definitionen zu verbessern und die Beobachtervereinbarung zwischen Nephropathologen weltweit auszubauen [8, 9]. Es wurden neue Definitionen für mesangiale Hyperzellularität, Adhäsion, fibrinoide Nekrose und für zelluläre, fibrozelluläre und fibröse Halbmonde fixiert [8]. Eine detaillierte Liste der im überarbeiteten ISN/RPS-Klassifizierungssystem vorgeschlagenen Änderungen und Empfehlungen ist in Tab. 2 aufgeführt. Auf der Grundlage des 1984 veröffentlichten Aktivitäts- und Chronifizierungsindex der National Institutes of Health (NIH) [10] wurde ein modifiziertes NIH-Scoring-System für die Aktivität und Chronifizierung von LN eingeführt (Tab. 3), um das Ausmaß der Gesamtaktivität und Chronifizierung semiquantitativ zu bewerten. Das Vorhandensein von endokapillärer Hyperzellularität, Neutrophilen/Karyorrhexis, fibrinoider Nekrose, hyalinen Ablagerungen, zellulären oder fibrozellulären Halbmonden innerhalb der Glomeruli sowie interstitieller Entzündungen innerhalb des Kortex wird je nach Prozentsatz der betroffenen Glomeruli mit Werten von 0 bis 3 bewertet. Die fibrinoide Nekrose wurde in eine eigenständige Kategorie umgewandelt, da die meisten Karyorrhexien durch den apoptotischen Zelltod von Neutrophilen verursacht werden [8]. Bei der Bewertung der Lupus-Chronizität wird der Begriff „globale Sklerose“ für Glomeruli verwendet, die vollständig sklerotisch sind, und jede andere Form der Glomerulosklerose sollte als Segmentsklerose betrachtet werden [8]. Die Entscheidung, ob global sklerotische Glomeruli durch Lupus oder durch eine nicht-lupusbedingte Schädigung, z. B. durch Arterionephrosklerose, entstanden sind, kann schwierig oder in einigen Fällen sogar unlösbar sein. Zu den histopathologischen Anzeichen in global sklerotischen Glomeruli, die auf LN hindeuten, gehören fragmentierter Tuft mit umliegender Fibrose und eine weitreichende Zerstörung der Bowman-Kapsel sowie Restablagerungen, die in der Immunfluoreszenzanalyse gefunden werden [8]. Die Einzelheiten des modifizierten NIH-Scoresystems für LN-Aktivität und Chronizität sind in Tab. 3 aufgeführt.

**Table 1:** 

Klasse	Diagnose	Definition
*I*	Minimale mesangiale LN	Normal bei Lichtmikroskopie (LM) mit mesangialen Ablagerungen bei Immunfluoreszenz (IF) oder Elektronenmikroskopie (EM)
*II*	Mesangial proliferative LN	Rein mesangiale Hyperzellularität bei LM mit mesangialen Ablagerungen bei IF; kann seltene subepitheliale oder subendotheliale Ablagerungen bei IF oder EM aufweisen (nicht bei LM)
*III*	Fokale LN	Aktive oder inaktive segmentale oder globale endokapilläre ± extrakapilläre GN bei LM in < 50 % der Glomeruli; gewöhnlich mit subendothelialen Ablagerungen
*IV*	Diffuse LN	Aktive oder inaktive segmentale oder globale endokapilläre extrakapilläre GN bei LM in ≥ 50 % der Glomeruli
*V*	Membranöse LN	Globale oder segmentale granuläre subepitheliale Ablagerungen entlang des GMB bei LM und IF oder EM; wenn Klasse III oder IV vorhanden ist, müssen bei > 50 % der Kapillaren von > 50 % der Glomeruli ± mesangialen Veränderungen auftreten
*VI*	Erweiterte Sklerose LN	≥ 90 % der glomerulären Sklerose ohne Restaktivität

**Table 2:** 

Kategorie	Empfehlungen
*Klasse II*	Mesangiale Hyperzellularität: ≥ 4 Mesangialzellen = vollständig von Matrix umgebene Kerne im mesangialen Bereich, ohne Hilärregion
*Klasse III und Klasse IV*	Endokapilläre Proliferation ersetzt durch endokapilläre Hyperzellularität Sichel = bestehend aus ≥ 2 Zellschichten und extrakapillärer Hyperzellularität, mit einer variablen Mischung von Zellen. Fibrin und fibröse Matrix können vorhanden sein – Zelluläre Sichel: > 75 % Zellen und Fibrin, < 25 % fibröses Gewebe – Fibrozellulärer Halbmond: 25–75 % Zellen und Fibrin, Rest fibröse Matrix – Fibröser Halbmond: > 75 % fibröse Matrix, < 25 % Zellen und Fibrin Adhäsion = Bereich isolierter Kontinuität des extrazellulären Matrixmaterials zwischen Tuft und Kapsel, selbst wenn das zugrunde liegende Segment keine Übersklerose aufweist Fibrinoidnekrose = Fibrin assoziiert mit einer Störung der glomerulären Basalmembran und/oder Lyse der Mesangialmatrix; Karyorrhexis nicht erforderlich Klasse IV: Eliminierung segmentaler und globaler Unterteilungen Einführung des NIH-Scoring-Systems für Aktivität und Chronizität anstelle der AC- und A/C-Parameter
*Tubulo-interstitielle Läsionen*	Geben Sie an, ob eine interstitielle Entzündung mit oder ohne interstitielle Fibrose auftritt

**Table 3:** 

**Modifizierter NIH-Aktivitätsindex**	**Definition**	**Score**
Endokapilläre Hyperzellularität	1+: < 25 %, 2+: 25–50 %, 3+: > 50 % Glomeruli	0–3
Neutrophile/Karyorrhexis	1+: < 25 %, 2+: 25–50 %, 3+: > 50 % Glomeruli	0–3
Fibrinoidnekrose	1+: < 25 %, 2+: 25–50 %, 3+: > 50 % Glomeruli	(0–3) × 2
Hyaline Ablagerungen	1+: < 25 %, 2+: 25–50 %, 3+: > 50 % Glomeruli	0–3
Zelluläre/fibro-zelluläre Halbmonde	1+: < 25 %, 2+: 25–50 %, 3+: > 50 % Glomeruli	(0–3) × 2
Interstitielle Entzündung	1+: < 25 %, 2+: 25–50 %, 3+: > 50 % im Cortex	0–3
*Gesamt*		*0–24*
**Modifizierter NIH-Chronizitätsindex**	**Definition**	–
Globale und/oder segmentale Sklerose	1+: < 25 %, 2+: 25–50 %, 3+: > 50 % Glomeruli	0–3
Fibröse Halbmonde	1+: < 25 %, 2+: 25–50 %, 3+: > 50 % Glomeruli	0–3
Röhrenatrophie	1+: < 25 %, 2+: 25–50 %, 3+: > 50 % im Cortex	0–3
Interstitielle Fibrose	1+: < 25 %, 2+: 25–50 %, 3+: > 50 % im Cortex	0–3
*Gesamt*		*0–12*

## Management

Die Endziele der LN-Behandlung sind die Prävention von CKD und Nierenerkrankungen im Endstadium (CKD G5), die Optimierung der Lebensqualität und die Verbesserung der Überlebensrate. Die Nierenbiopsie ist der Eckpfeiler der aktuellen Standardpraxis, basierend auf den prognostischen Werten der histopathologischen Befunde (d. h. histologische Klasse, Aktivitäts- und Chronizitätsindizes, Vorhandensein von Halbmondläsionen und/oder tubulointerstitiellen Läsionen), und die Behandlung variiert je nach Schweregrad der Erkrankung und Risiko für fortschreitende Nierenschäden. Patient*innen mit aktiver, schwerer proliferativer oder membranöser LN (Klasse III, IV und V mit Proteinurie im nephrotischen Bereich) benötigen eine immunsuppressive Behandlung, um einen fortschreitenden und irreversiblen Nephronverlust zu vermeiden. Die Therapie gliedert sich in der Regel in eine Induktions- und eine Erhaltungstherapie und soll ein vollständiges klinisches Ansprechen erreichen. Im Gegensatz dazu basiert die Behandlung von Patient*innen mit Klasse I/II (oder nicht-nephrotischer reiner LN der Klasse V) hauptsächlich auf konservative Maßnahmen. Bei Patient*innen mit LN der Klasse VI liegt eine ausgedehnte chronische histologische Schädigung oder CKD G5 vor, daher ist eine immunsuppressive Therapie nur bei ausgeprägter extra-renaler Aktivität indiziert. In diesem Abschnitt werden die Indikationen und Ziele der LN-Behandlung erörtert und aktuelle Behandlungsempfehlungen auf der Grundlage der derzeit verfügbaren Literatur gegeben.

## Definition des Therapie-Ansprechens

Es wurden mehrere nationale und internationale Leitliniendefinitionen veröffentlicht, die sich auf die Behandlung der LN beziehen [11–15]. Da ein signifikanter Anteil der LN-Patient*innen innerhalb von 10 Jahren nach der Diagnose eine CKD im Stadium 5 entwickelt [16], ist die Identifizierung klinischer Prädiktoren für das langfristige renale Ergebnis von wesentlicher Bedeutung. In den meisten klinischen Studien wurden der Serumkreatininspiegel, die Proteinausscheidung im Urin und das Vorliegen einer glomerulären Hämaturie zur Beurteilung des Ansprechens auf die Therapie herangezogen.

Daten aus den Studien Euro-Lupus Nephritis (ELNT) und MAINTAIN ergaben, dass ein Grenzwert für die Proteinurie von < 0,8 bzw. < 0,7 g/Tag nach 12 Monaten mit dem besten langfristigen Nierenergebnis assoziiert ist (d. h. Serumkreatinin < 1,0 mg/dl nach 7 Jahren) [17, 18]. Diese Beobachtung wird auch durch weitere Daten aus klinischen Studien gestützt [19], während diese Ergebnisse größtenteils mit den Daten aus der realen Beobachtung von Patient*innen mit schwerer LN in ethnisch unterschiedlichen Gruppen übereinstimmen [20–23]. Allerdings spiegelt das Ausmaß der Proteinurie nicht immer die histologische Aktivität wider und kann auf irreversible interstitielle Veränderungen ohne Krankheitsaktivität hinweisen [24, 25]. Trotz der Bedeutung der glomerulären Hämaturie als diagnostischem Surrogatmarker führte ihre Umsetzung in die Vorhersagemodelle nicht zu einer besseren Vorhersage der Progredienz der Grunderkrankung [17, 21, 26]. Der Einsatz wiederholter per-Protocol-Nierenbiopsien bei der Therapiebeurteilung wird in der wissenschaftlichen Gemeinschaft intensiv diskutiert und prospektive Studien laufen derzeit [27, 28].

In der aktuellen klinischen Praxis sollte das Ansprechen auf die Behandlung als vollständig (CR), partiell (PR) und kein Ansprechen definiert werden, das 6–12 Monate nach Behandlungsbeginn in Abhängigkeit vom Vorliegen einer Proteinurie im nephrotischen Bereich beurteilt wird.

Wir empfehlen die Anwendung der folgenden Kriterien für das klinische Ansprechen auf der Grundlage der Empfehlungen von EULAR/European Renal Association (ERA) [13]. Im Gegensatz zu diesen Empfehlungen sind wir der Ansicht, dass die UPCR oder UACR ausreicht, um das klinische Ansprechen zu ermitteln, und dass sie für die klinische Routine besser geeignet ist als die Messung der 24 h-Proteinurie:

- Vollständiges klinisches Ansprechen: UPCR < 0,5–0,7 g/g bis 12 Monate nach Therapiebeginn
- Partielles klinisches Ansprechen: Verbesserte Proteinurie-Werte innerhalb von 3 Monaten und eine 50 %ige Reduktion der Proteinurie innerhalb von 6 Monaten nach Therapiebeginn
- Kein Ansprechen: Keine CR oder PR erreicht

Da sich die Proteinurie bei Patient*innen mit einer Proteinurie im nephrotischen Bereich zu Beginn der Therapie langsamer erholen kann, sind bei dieser Patientenpopulation weitere 6–12 Monate zur Beurteilung des klinischen Ansprechens erforderlich [29].

## Behandlung von nicht-proliferativen Formen der LN

**Figure 1:**
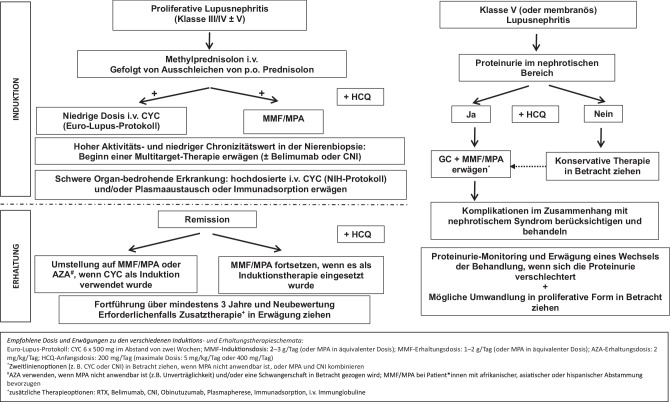

Die Behandlung von Patient*innen mit LN der Klassen I/II (wenn keine Lupus-Podozytopathie vorliegt) und V mit Proteinurie im subnephrotischen Bereich und normaler GFR konzentriert sich im Allgemeinen auf konservative Maßnahmen, einschließlich Blutdruckkontrolle (d. h. Blockade des Renin-Angiotensin-Aldosteron-Systems – RAS) und Immunmodulation mit Malariamitteln (d. h. Hydroxychloroquin – HCQ). Die immunsuppressive Behandlung wird angewendet, um extrarenale Manifestationen zu kontrollieren. In diesem Abschnitt werden der Einsatz von Antimalariamitteln und die Behandlung von LN der Klasse V näher erläutert (siehe auch Abb. 1), während zusätzliche konservative Behandlungsmöglichkeiten an anderer Stelle zusammengefasst werden.

### Malariamittel

Unter den Malariamitteln ist HCQ (auch bekannt als Hydroxychloroquinsulfat) ein etabliertes und weit verbreitetes immunmodulierendes Medikament in der Behandlung des SLE [30]. In der LN haben mehrere Beobachtungsstudien einen Nutzen in der Verbesserung der renalen Ansprechrate, der Verringerung des Risikos eines Rezidivs und der Verhinderung des Fortschreitens einer CKD gezeigt, allerdings ist die Datenlage begrenzt [19]. Die Anwendung von HCQ bei Patient*innen mit LN (jeder Klasse, außer wenn kontraindiziert) wird für alle LN-Patient*innen empfohlen. Die Dosierung wird entsprechend den Empfehlungen des Herstellers empfohlen. In einer retrospektiven Analyse wurde festgestellt, dass ein HCQ-Mindestblutspiegel von 0,6 mg/l mit einer geringeren Rate von renalen Rezidiven einhergeht und dazu beiträgt, Patient*innen mit mangelnder Therapiecompliance zu erkennen [31]. Daher kann eine Überwachung des HCQ-Blutspiegels nützlich sein, sofern verfügbar.

Netzhauttoxizität ist eine bekannte Nebenwirkung der Langzeitanwendung von HCQ [32]. Daher sollten sich die Patient*innen einer Ausgangsuntersuchung und einer regelmäßigen (5 Jahren nach Therapiebeginn und in weiterer Folge jährlichen) augenärztlichen Untersuchung unterziehen.

### Behandlung der reinen Klasse-V-LN

Klasse V (auch als membranöse LN bezeichnet) ist eine besondere Form der LN, die durch subepitheliale Immunkomplexablagerungen gekennzeichnet ist. Neuere internationale Behandlungsempfehlungen beruhen weitgehend auf Daten aus Beobachtungsstudien oder kleinen RCTs, die häufig Patient*innen mit gemischt proliferativen Fällen einschlossen [33, 34]. Die Analyse klassenspezifischer und langfristiger Nachbeobachtungsdaten aus neueren RCTs ist noch nicht abgeschlossen und wird weiterführende Informationen über spezifischere Behandlungsoptionen für diese Patientengruppe liefern [34].

Die Erstbehandlung richtet sich nach dem Ausmaß der Proteinurie. Patient*innen mit Proteinurie im subnephrotischen Bereich haben häufig einen gutartigen Krankheitsverlauf und benötigen unter Umständen keine spezifische immunsuppressive Therapie im Hinblick auf die Nierenbeteiligung. Andererseits profitieren Patient*innen mit Proteinurie im nephrotischen Bereich (> 3 g/g) von einer immunsuppressiven Therapie [33, 35, 36]. Die immunsuppressive Medikation sollte auf die individuellen Bedürfnisse der Patient*innen unter Berücksichtigung der extrarenalen Manifestationen abgestimmt werden. Die Wirksamkeit von Mycophenolatmofetil (MMF), Cyclosporin und Cyclophosphamid (CYC) ist weitgehend belegt [37, 38], während Rituximab (RTX) und Tacrolimus (TAC) ebenfalls zur Verringerung der Proteinurie in Betracht gezogen werden können [39–42]. Glukokortikoide (GC) sollten zu jedem dieser therapeutischen Szenarien hinzugefügt werden (siehe Abb. 1). Unabhängig vom Ausmaß der Proteinurie sollten allen Patient*innen konservative Interventionen angeboten werden (d. h. Blutdruckkontrolle, Prävention und Behandlung von Komorbiditäten), und Komplikationen im Zusammenhang mit einer Proteinurie im nephrotischen Bereich (d. h. Thrombose, Dyslipidämie, Ödem) müssen beachtet werden. Im Gegensatz dazu müssen Patient*innen mit einer proliferativen Komponente (gemischt proliferative und membranöse Fälle) entsprechend den Empfehlungen für proliferative LN behandelt werden, wie im nächsten Abschnitt beschrieben. Der therapeutische Ansatz bei Patient*innen mit reiner Klasse-V-LN ist in Abb. 1 zusammengefasst.

### Proliferative LN: Induktionsbehandlung mit Immunsuppressiva

Proliferative Formen von LN (Klasse III/IV ± Klasse V) erfordern eine immunsuppressive Therapie, da unerwünschte renale Ereignisse in diesen Patientengruppen am häufigsten auftreten. Ziel der Induktionstherapie ist die sofortige Abschwächung der Inflammation im Gewebe und die Unterdrückung von Autoimmunprozessen [43, 44]. Das Behandlungsziel ist, innerhalb von 3 bis 6 Monaten nach Therapiebeginn eine Remission zu erreichen.

Intravenöse Glukokortikoide (GC) (0,25–0,5 g über 1–3 Tage), gefolgt von einer möglichst niedrigen Anfangsdosis von oralem Prednison sind die Eckpfeiler der Induktionstherapie (siehe Tab. 4). Da neuere Studien darauf hinweisen, dass eine reduzierte GC-Exposition eine zuverlässige Option ist, um eine Remission mit weniger therapiebedingten Nebenwirkungen zu erreichen [34], empfehlen wir nach Möglichkeit niedrig dosierte GC-Schemata.

**Table 4:** 

	Standard	Mäßig	Verringert
Methylprednisolon i.v. (pulsieren)	Null oder 0,25–0,5 g/Tag^a^	0,25–0,5 g/Tag^a^	**0,25–0,5** **g/Tag**^a^
*Prednisonäquivalent zum Einnehmen*			
Woche 1–2	0,8–1,0 mg/kg^b^	0,6–0,7 mg/kg^c^	**0,5–0,6** **mg/kg**^d^
Woche 3–4	0,6–0,7 mg/kg	0,5–0,6 mg/kg	**0,3–0,4** **mg/kg**
Woche 5–6	30 mg	20 mg	**15** **mg**
Woche 7–8	25 mg	15 mg	**10** **mg**
Woche 9–10	20 mg	12,5 mg	**7,5** **mg**
Woche 11–12	15 mg	10 mg	**5** **mg**
Woche 13–14	12,5 mg	7,5 mg	**2,5** **mg**
Woche 15–16	10 mg	7,5 mg	**2,5** **mg**
Woche 17–20	7,5 mg	5 mg	**2,5** **mg**
Woche 21–24	5 mg	< 5 mg	**2,5** **mg**
> 25 Wochen	< 5 mg	< 5 mg	**2,5** **mg**
> 52 Wochen	Absetzen erwägen, wenn keine renale und extrarenale Aktivität vorhanden ist		

^a^Gepulste i.v. Methylprednisolon-Dosen bis zu 3 Tage; orale GC basierend auf täglicher Anwendung

^b^Maximal 80 mg

^c^Maximal 50 mg

^d^Maximal 40 mg

Als tragende Säule der Therapie wird die zusätzliche Gabe von oralem MMF (2–3 g/Tag oder Mycophenolsäure [MPA] in einer äquivalenten Dosis über 6 Monate) oder niedrig dosiertem intravenösem CYC (Euro-Lupus-Schema: 6 Mal 500 mg alle 2 Wochen) zu GC empfohlen. Selbstverständlich muss die Wahl des Immunsuppressivums auf die Merkmale und Präferenzen der Patient*innen abgestimmt werden, insbesondere auf die ethnische Zugehörigkeit (MMF/MPA wird bei Patient*innen mit afrikanischen, asiatischen und hispanischen Abstammungen bevorzugt), frühere Medikamentenexposition (z. B. CYC), schwere, organbedrohende Erkrankungen (hochdosiertes intravenöses CYC gemäß NIH-Protokoll – siehe Abb. 1), Risiko für Unfruchtbarkeit und Medikamentenadhärenz. Eine Kombinationstherapie mit GC, MMF/MPA oder CYC in Kombination mit CNI oder Belimumab sollte insbesondere bei Patient*innen mit einer hohen Aktivität/niedrigen Chronizität in der Nierenbiopsie (Abb. 1) in Betracht gezogen werden, da dieser Ansatz zu höheren Remissionsraten führt [45].

Diese Überlegungen beruhen auf Daten aus kürzlich durchgeführten randomisierten klinischen Studien, in denen neue therapeutische Wirkstoffe getestet wurden, die in Zukunft als zusätzliche Erstlinienbehandlung in Frage kommen könnten. Voclosporin (ein oraler CNI der neuen Generation – zweimal täglich 23,7 mg) in Kombination mit MMF und GC in reduzierter Dosierung zeigte signifikant verbesserte Remissionsraten (basierend auf Veränderungen im Ausmaß der Proteinurie) im Vergleich zur Standardtherapie (GC + MMF) bei Patient*innen mit aktiver proliferativer LN (AURORA 1‑Studie). Der gleiche Nutzen von Voclosporin wurde auch bei LN-Patient*innen beobachtet, deren Erkrankung mehr als sechs Monate vor Therapiebeginn auftrat, sowie bei Patient*innen mit schwerer LN (definiert als UPCR > 3/g zu Studienbeginn) [46]. Um die B‑Zell-Reaktionen bei LN-Patient*innen zu beeinflussen, bildet die Hemmung des B‑Lymphozyten-Stimulators (Blys) die Grundlage der Therapie mit Belimumab. Der Einsatz dieses Wirkstoffs führte zu höheren Ansprechraten (definiert als PCR < 0,7 g/g, eine eGFR, die nicht schlechter als 20 % unter dem Ausgangswert lag oder mindestens 60 ml/min pro 1,73 m^2^ betrug, und kein Therapieversagen) im Vergleich zu Placebo, wenn er zur Standardtherapie hinzugefügt wurde [47].

**Figure 2:**
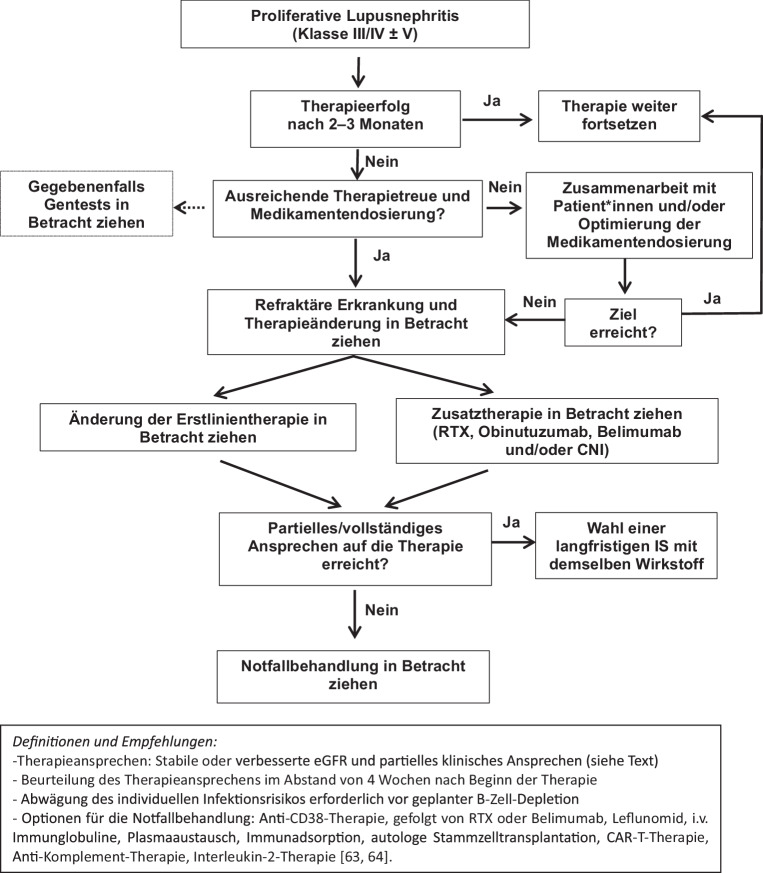

B‑Zell-depletierende Wirkstoffe mittels anti-CD20-Antikörpern sind bei der Behandlung von LN umfassend untersucht worden [48]. Die LUNAR-Studien waren die größten randomisierten kontrollierten Rituximab-RTX-Studien (RCT) und haben den primären Endpunkt des Nierenansprechens in Woche 52 bei LN leider nicht erreicht [49, 50]. Dennoch zeigten Daten aus Praxisstudien, dass bestimmte Patientengruppen (d. h. Patient*innen mit refraktärer oder rezidivierender Erkrankung) von der Anwendung von RTX (entweder allein oder in Kombination) profitieren könnten [13, 14]. Darüber hinaus zeigte eine Post-hoc-Analyse der LUNAR-Studie eine höhere renale Ansprechrate bei LN-Patient*innen, die bis 365 Tage nach Therapiebeginn eine vollständige CD19-Zelldepletion erreicht hatten [51]. Daher wird bei Verwendung von RTX eine Analyse der CD19-Zellen empfohlen. Der Einsatz des humanisierten monoklonalen Anti-CD20-Antikörpers Obinutuzumab führte in einer Phase-II-Studie (NOBILITY, NCT02550652) im Vergleich zum Kontrollarm zu einer höheren Ansprechrate der LN bis Woche 52 ohne signifikante Sicherheitssignale in Bezug auf infektiöse Komplikationen [52] Zusammengefasst kann eine B‑Zell-Depletion mittels eines anti-CD20-Antikörpers insbesondere als Zusatzstrategie bei Patient*innen mit refraktärer LN herangezogen werden (Abb. 2).

Bei LN-Patient*innen mit schwerer organbedrohender Erkrankung kann eine hochdosierte CYC-Behandlung nach dem NIH-Protokoll (0,5–1 g/m^2^ monatlich über 6 Monate) und zusätzlich ein Plasmaaustausch oder eine Immunadsorption in Betracht gezogen werden. Ein Überblick über die empfohlene Induktionstherapie bei proliferativer LN ist in Abb. 1 dargestellt.

Ziel der Erhaltungstherapie ist es, dass nach der Induktionsphase erreichte Ansprechen zu konsolidieren. Optimale Erhaltungstherapien zur Remissionserhaltung sollten auf individuellen patientenbezogenen Faktoren und Präferenzen basieren, z. B. vorangegangene Therapien, Komorbiditäten, ethnische Zugehörigkeit, Unfruchtbarkeitsrisiko oder Therapieadhärenz. Im Allgemeinen sollte MMF/MPA die Grundlage der Erhaltungstherapie bilden, während Azathioprin (AZA) und CNI bei Vorliegen von Kontraindikationen eingesetzt werden können.

Patient*innen, die eine MMF/MPA-Induktionstherapie erhalten haben, sollten weiterhin denselben Wirkstoff in einer Dosierung von 1–2 g/Tag (oder MPA in äquivalenter Dosis) als Erhaltungsdosis erhalten. Wenn CYC als Induktionstherapie angewendet wurde, wird eine Umstellung auf MMF/MPA oder AZA (2 mg/kg/Tag) empfohlen. Zu beachten ist, dass AZA bei Patientinnen als Erstlinientherapie empfohlen wird, wenn MMF/MPA kontraindiziert ist oder eine Schwangerschaft vorliegt bzw. geplant wird (Abb. 1). Im Allgemeinen sollte die Dauer der immunsuppressiven Erhaltungstherapie mindestens drei Jahre lang beibehalten werden, bevor ein langsames und schrittweises Absetzen in Erwägung gezogen und individuell angepasst wird [53]. Wenn Belimumab oder CNI zur Induktion einer Remission eingesetzt wurden, könnten sie als Teil der Erhaltungstherapie zur Remissionserhaltung in Betracht gezogen werden. Es fehlen jedoch Daten über den langfristigen Einsatz von Belimumab als Erhaltungstherapie. Demgegenüber zeigte die AURORA-2-Fortsetzungsstudie zu Voclosporin eine stabile eGFR und Proteinurie sowie ein geringeres Risiko für Relapse der Lupusnephritis drei Jahre nach Therapiebeginn (Teng Y.K. et al., vorgestellt auf dem 59th European Renal Association (ERA) Congress 2022, Paris. Abstract FC 054). Darüber hinaus wurde gezeigt, dass Belimumab das Risiko eines Krankheitsschubs reduziert, während CNI in erster Linie antiproteinurische Eigenschaften aufweist und daher beide Wirkstoffe das Fortschreiten der CKD bei LN verlangsamen könnten [34, 46, 47, 54]. Die Empfehlung zur Erhaltungstherapie zur Remissionserhaltung ist in Abb. 1 dargestellt.

Eine Reduktion der Kortikosteroiddosis auf die „niedrigstmögliche Dosis“ sollte angestrebt werden. Zudem kann auch ein Absetzen der Kortikosteroide je nach renaler und extrarenaler SLE Aktivität in Betracht gezogen werden [12]. Sollte eine längere Anwendung erforderlich sein, ist eine Prednison-Dosis ≤ 5 mg/Tag mit einem geringen Risiko für Folgekomplikationen (d. h. Infektionen, Herz-Kreislauf-Erkrankungen, Osteoporose und Stoffwechselveränderungen) assoziiert und wird empfohlen [55]. Bestimmte Therapiestrategien, wie die ergänzende Therapie mit Belimumab, CNI oder CD20-depletierenden monoklonalen Antikörpern, können ein schnelleres Ausschleichen und Absetzen von GC ermöglichen [47, 52, 56, 57].

### Behandlung von nicht ansprechender und refraktärer Erkrankung

Eine refraktäre Erkrankung (RD) ist definiert als ein Ausbleiben des klinischen Ansprechens nach geeigneter Induktionstherapie, es gibt jedoch keine genaue globale Definition [58]. Spezifische Empfehlungen zur Beurteilung des klinischen Ansprechens sind bereits im Abschnitt „Definition von Ansprechen auf die Therapie“ aufgeführt. Der Zeithorizont, um eine RD nach Induktionstherapie in Betracht zu ziehen, variiert zwischen den EULAR/ERA- und den ACR-Leitlinien [13, 14]. Wir empfehlen, alle 4 Wochen eine klinische Bewertung vorzunehmen, und bei einem Therapieversagen kann bereits 6 Monate nach Therapiebeginn eine RD in Betracht gezogen werden. Im Allgemeinen können alle Erstlinienmedikamente zur Behandlung einer RD eingesetzt werden. Darüber hinaus könnten bestimmte Notfalltherapien (insbesondere RTX, Voclosporin, Belimumab oder Obinutuzumab ± GCs) zu einer Verringerung der Krankheitsaktivität führen, es fehlt jedoch eine umfassende Evidenz zur Wirksamkeit dieser Wirkstoffe bei einer RD. Wenn alle diese Therapiemöglichkeiten ausgeschöpft sind, liegen nur begrenzte Daten zu weiteren Substanzen (siehe Abb. 2) vor, die an anderer Stelle nachgelesen werden können [58, 59]. Wichtig ist, dass insbesondere mangelnde Therapietreue und eine angemessene Dosierung der laufenden Therapie überprüft und bedacht werden müssen, da diese Faktoren häufig auftreten und zu einem insuffizienten Ansprechen auf die Therapie führen. In diesen Fällen kann eine Messung der Wirkstoffspiegel oder eine Bewertung der biologischen Wirkungen der Immunsuppressiva (falls zutreffend) hilfreich sein. In einigen klinischen Szenarien können wiederholte Nierenbiopsien dazu beitragen, Patient*innen mit Progressionsrisiko zu identifizieren, sowie solche, die eine Anpassung der Therapie benötigen. Dennoch sollten alle Patient*innen, die nach einer geeigneten immunsuppressiven Behandlung kein klinisches Ansprechen zeigen, an ein Fachzentrum überwiesen werden. Die Empfehlung zur Diagnose und Behandlung einer RD ist in Abb. 2 zusammengefasst.

### Konservative Therapie und Behandlung von Komorbiditäten

Trotz erheblicher Verbesserungen in der Therapie, die zu einer besseren Kontrolle der Krankheit geführt haben, sind Patient*innen mit LN nach wie vor einem hohen Mortalitätsrisiko ausgesetzt, das insbesondere durch kardiovaskuläre und infektiöse Komplikationen sowie durch Malignome bedingt ist [60–62]. Die Prävention von Organschäden und eine angemessene Behandlung von Komorbiditäten sind unerlässlich, um die durch die Krankheit und immunsuppressive Therapie bedingte Mortalität in dieser Patientengruppe zu minimieren. Die Strategien zur Kontrolle von Komorbiditäten und therapiebedingten Komplikationen sind in Tab. 5 zusammengefasst.

**Table 5:** 

Schwerpunkt	Beschreibung	Empfehlungen	
Unterstützende Therapieoptionen und allgemeine Empfehlungen	Allen LN-Patient*innen sollten zusätzlich zur IS-Therapie diese allgemeinen Behandlungsoptionen angeboten werden. Bei der Behandlung von Patient*innen mit LN Klasse II und subnephrotischer Proteinurie und normaler GFR der Klasse II (ohne das Vorliegen einer Lupus-Podozytopathie) und bei der LN Klasse V ohne große Proteinurie liegt der Schwerpunkt im Allgemeinen auf konservativen Interventionen	1.	Alle Patient*innen sollten zu allgemeinen Maßnahmen zum Schutz der Nieren aufgeklärt werden
		2.	Aufgrund ihrer antiproteinurischen und antihypertensiven Wirkung wird bei allen nicht schwangeren LN-Patientinnen eine Blockade des Renin-Angiotensin-Aldosteron-Systems empfohlen
		3.	Alle Patient*innen sollten Malariamittel (d. h. HCQ) erhalten
Infektionen	Personen mit LN haben ein höheres Erkrankungs- und therapiebedingtes Infektionsrisiko. Nierenbeteiligung, systemische Therapien, Krankheitsaktivität und schwere Leukopenie gelten als unabhängige Risikofaktoren für infektiöse Komplikationen	1.	Das Infektionsrisiko sollte vor Beginn der systemischen Therapie beurteilt und während der Behandlung aktiv überwacht werden
		2.	Alle Patient*innen sollten vor Beginn einer immunsuppressiven Therapie auf latente Tuberkulose, HBV und HCV untersucht werden
		3.	Eine prophylaktische Anwendung von niedrig dosiertem TMP/SMX oder eine alternative Anwendung von Atovaquon oder monatlicher aerosolierter Pentamidin-Behandlung zur Vermeidung einer Pneumocystis-jirovecii-Pneumonie (oder Harnwegs- und anderen Atemwegsinfektionen) kann nach einer tiefgreifenden Risiko-Nutzen-Stratifizierung bei Patient*innen mit hohem Infektionsrisiko (d. h. bei Patient*innen, die hochdosierte Kortikosteroide erhalten) individuell in Betracht gezogen werden
		4.	Die Anwendung von GC sollte minimiert werden, um schwere infektiöse Komplikationen zu vermeiden
		5.	Eine Impfung sollte nach Berücksichtigung möglicher Kontraindikationen durchgeführt werden, vor allem die jährliche Grippeimpfung und Impfung gegen Pneumokokken gemäß nationalen Empfehlungen. Die Häufigkeit der COVID-19-Impfung muss bestimmt werden. Abgeschwächte Lebendimpfstoffe sollten generell vermieden werden (in Einzelfällen kann eine Herpes-zoster-Impfung erwogen werden)
aPL-Positivität/APS	aPL-Positivität und APS bei LN sind schlechte prognostische Faktoren, die mit einem höheren Risiko für thrombotische Komplikationen (d. h. Nierenarterien‑/Venenthrombose, thrombotische Mikroangiopathie-Läsionen in Nierenbiopsien oder Allotransplantatthrombose nach Nierentransplantation) und einem schlechten renalen Ergebnis verbunden sind	1.	Bei LN-Patient*innen mit aPL-positiver Einstellung nach einer Beurteilung des Blutungsrisikos könnte eine niedrig dosierte Acetylsalicylsäure in Betracht gezogen werden, wobei empfohlen wird, während der Schwangerschaft vor der 16. Schwangerschaftswoche zu beginnen
		2.	Eine zusätzliche Antikoagulanzien-Behandlung (d. h. niedermolekulares Heparin) kann bei LN-Patient*innen mit aPL-positiver Wirkung oder APS während der Schwangerschaft oder postoperativ oder bei Patient*innen mit aPL-Profil mit hohem Risiko in Betracht gezogen werden
		3.	Bei LN-Patient*innen mit APS, die ein Nierentransplantat erhalten haben, wird eine Antikoagulationsbehandlung (d. h. niedermolekulares Heparin) empfohlen
Kardiovaskuläre Risiken	Personen mit LN haben ein höheres Risiko für Herz‑/Kreislauferkrankungen als die Allgemeinbevölkerung. Die Krankheit selbst, unterschiedliche Therapieformen und klassische Risikofaktoren tragen zu diesem Risiko bei	1.	Ein Screening auf Hyperlipidämie wird empfohlen; die üblichen Leitlinien zur Prävention und Behandlung sollten befolgt werden
		2.	Ein Screening auf arterielle Hypertonie wird empfohlen; die üblichen Leitlinienlinien zur Prävention und Behandlung sollten befolgt werden
		3.	Ein Screening auf Prädiabetes und manifesten DM wird empfohlen; die üblichen Leitlinien zur Prävention und Behandlung sollten befolgt werden
		4.	Eine Raucherentwöhnung und regelmäßige körperliche Betätigung wird empfohlen
		5.	Die Anwendung von GC sollte bei Patient*innen mit kardiovaskulären Risikofaktoren auf ein Minimum beschränkt werden
		6.	Die Anwendung von SGLT2-Inhibitoren sollte in Betracht gezogen werden, wenn eine CKD vorliegt und keine Kontraindikationen vorliegen. Zulassungsüberschreitende Anwendung; die üblichen CKD-Richtlinien sollten befolgt werden
Osteoporose	Personen mit LN haben ein höheres Osteoporose-Risiko, das sowohl durch krankheitsbedingte als auch durch nicht krankheitsbedingte Faktoren verursacht wird	1.	Allen Patient*innen, die eine GC-Behandlung beginnen oder langfristig erhalten, wird eine Beurteilung des Frakturrisikos mit Messung der BMD und Verwendung des FRAX-Risikorechners empfohlen
		2.	Alle Patient*innen, die eine GC-Behandlung beginnen oder langfristig erhalten, sollten über Strategien zur Verbesserung der Ernährung (d. h. Vitamin-D- und Kalziumaufnahme), zur Verringerung des Sturzrisikos und zu anderen Änderungen der Lebensweise beraten werden. Bezüglich der Prävention sind die üblichen Leitlinien zu befolgen
		3.	Alle Patient*innen mit erhöhtem Frakturrisiko sollten eine geeignete pharmakologische Behandlung erhalten. Bezüglich der Behandlung sind die üblichen Leitlinien zu befolgen. Östrogenhaltige Substanzen sollten vermieden werden, insbesondere bei Patient*innen mit aPL-positiver Wirkung und Krankheitsaktivität
		4.	Zur Vermeidung von Knochenschwund sollte die Anwendung von GC auf ein Minimum reduziert werden

*ACEI* Angiotensin-Converting-Enzym-Hemmer, *aPL* Antiphospholipid, *APS* Antiphospholipid-Syndrom, *AZA* Azathioprin, *BMD* Knochenmineraldichte, *CKD* Chronische Nierenerkrankung, *KVE* Kardiovaskuläre Erkrankung, *DM* Diabetes mellitus, *GC* Glukokortikoid, *HBV* Hepatitis-B-Virus, *HCV* Hepatitis-C-Virus, *HCQ* Hydroxychloroquin-Immunsuppression, *SGLT2* Natrium-Glucose-Transportprotein 2, *TMP/SMX* Trimethoprim-Sulfamethoxazol

### Behandlung terminaler Nierenerkrankung bei Patient*innen mit LN

Trotz wirksamer immunmodulatorischer Mittel entwickelt sich bei einem erheblichen Anteil der LN-Patient*innen eine CKD Stadium 5, die eine Nierenersatztherapie erfordert [2]. Dialysierte SLE-Patient*innen haben unabhängig von der Dialyseart eine vergleichbare Gesamtmortalität wie Patient*innen, die aufgrund von Nierenversagen anderer Ursachen eine Nierenersatztherapie erhalten [63, 64]. Frühe Daten deuten auf eine schlechtere Gesamtmortalitätsrate bei Patient*innen unter Peritonealdialyse (PD) im Vergleich zu Patient*innen mit Hämodialyse (HD) hin, die hauptsächlich auf technisches Versagen und infektiöse Komplikationen zurückzuführen war [65]. Eine neuere Metaanalyse, bei der Beobachtungsdaten verglichen wurden, ergab jedoch bessere kardiovaskuläre Ergebnisse bei SLE-Patient*innen, die mit PD behandelt wurden [66]. Wichtig ist, dass Patient*innen, die mit Antiphospholipid-Antikörpern behandelt werden, ein höheres Risiko haben, eine Thrombose des Gefäßzugangs zu entwickeln [16] und eine Antikoagulationstherapie erhalten sollten. Wir empfehlen, dass die Modalität der Nierenersatztherapie auf einer individualisierten Abwägung von Nutzen und Risiken basieren sollte.

Bei SLE-Patient*innen, die eine Nierenersatztherapie erhalten, scheint sich die Krankheitsaktivität nach Beginn der Nierenersatztherapie trotz fortbestehender serologischer Anomalien zu bessern [63, 67]. Trotzdem gibt es Unterschiede im klinischen Verlauf nach Einleitung der Nierenersatztherapie [68]. Kommt es zu einem Krankheitsschub, wird eine geeignete immunsuppressive Therapie empfohlen. Bei fehlender klinischer Aktivität kann eine Nierentransplantation angeboten werden, wie im nächsten Abschnitt erörtert.

## Nierentransplantation

Eine Nierentransplantation ist eine der besten Therapieoptionen für Patient*innen mit CKD Stadium 5. Es gibt keine evidenzbasierten Richtlinien, wie lange Patient*innen mit einer CKD Stadium 5 aufgrund einer LN bis zur Nierentransplantation warten sollten. Da das Vorliegen einer serologischen Krankheitsaktivität zum Zeitpunkt der Transplantation nicht mit Transplantationsergebnissen korreliert [69], empfehlen wir, eine Nierentransplantation bei stabilen Patient*innen mit CKD Stadium 5 aufgrund einer LN so früh wie möglich durchzuführen. Induktions- und Erhaltungstherapie nach einer Nierentransplantation entsprechen der bei anderen Nieren-transplantierten Patient*innen. Im Rahmen einer Nierentransplantation bei Patient*innen mit einer CKD Stadium 5 aufgrund einer LN sollten steroidsparende Behandlungsschemata mit Vorsicht angewendet werden. Im Falle eines Antiphospholipid-Syndroms (APS) sollten die Patient*innen während und nach der Transplantation mit Antikoagulanzien behandelt werden.

Die Rezidivrate der LN im Nierentransplantat liegt zwischen 2 und 11 %, und der Transplantatverlust aufgrund einer relapsierten LN ist sehr gering (2–4 % über 5–10 Jahre). Ebenso ist die SLE Relaps-Rate sehr gering (etwa 6 %), wahrscheinlich aufgrund der kontinuierlichen Immunsuppression, die nach Transplantation nötig ist [70, 71]. Die Diagnose einer relapsierten LN wird entsprechend der Vorgehensweise zur Diagnose einer LN in nativen Nieren durchgeführt. Die Behandlungsoptionen ähneln den in den Abschnitten zur Therapie beschriebenen immunsuppressiven Therapien. Die Patient*innen sollten an spezialisierte Zentren überwiesen werden, um die jeweilige Behandlung und Nachsorge durchzuführen.

## Schwangerschaft

Bevor SLE-Patientinnen versuchen, schwanger zu werden, sollte der SLE sechs Monate lang unter schwangerschaftsverträglichen Medikamenten in Remission sein. Ein aktiver SLE zum Zeitpunkt der Empfängnis ist ein starker Prädiktor für unerwünschte Schwangerschaftsausgänge. Wichtig ist, dass MMF mindestens 6 Monate vor der Empfängnis abgesetzt werden muss. HCQ, AZA, Cyclosporin A, TAC sowie Steroide sind Optionen zur Behandlung von Patientinnen mit SLE während der Schwangerschaft. CD20-depletierende Therapien können in sorgfältiger Risikostratifizierung auch während der Schwangerschaft eingesetzt werden. Da SLE-Schübe und Präeklampsie bei schwangeren SLE-Patientinnen häufig auftreten, müssen sie in einer Klinik, die für die Behandlung von Hochrisikoschwangerschaften spezialisiert ist, sorgfältig und multidisziplinär kontrolliert werden [2]. Wir empfehlen, schwangere SLE-Patientinnen vor der 16. Schwangerschaftswoche mit Aspirin 100 bis 150 mg/Tag zu behandeln, um eine Präeklampsie zu verhindern. Im Falle eines APS kann eine Antikoagulation mit niedermolekularem Heparin erforderlich sein. Darüber hinaus wird eine regelmäßige Bestimmung der Anti-Ro/SSA- und Anti-La/SSB-Antikörper empfohlen, da das Vorhandensein dieser Antikörper mit einem höheren Risiko für kongenitale und neonatale Komplikationen verbunden ist [72].

## Schlussfolgerungen und künftige Orientierungen

Insgesamt haben sich die Behandlungsmöglichkeiten des SLE in den letzten Jahren deutlich verbessert und bieten nun mehr Optionen für die Behandlung von LN-Patient*innen. Die Nierenbiopsie mit Aktivitäts- und Chronifizierungsscoring ist entscheidend für eine angemessene Therapie. Während die LN-Klassen I und II meist nur eine konservative Therapie benötigen, ist bei den LN-Klassen III und IV und teilweise bei Klasse V eine immunsuppressive Behandlung erforderlich. Die immunsuppressive Induktionstherapie umfasst MMF oder niedrig dosiertes CYC sowie eine Steroidbehandlung (in möglichst niedriger Dosis). Bei hohen Aktivitätswerten in der Nierenbiopsie kann zusätzlich eine CNI- oder Belimumab-Therapie in Betracht gezogen werden. CD20-depletierende Wirkstoffe bieten zusätzliche Alternativen bei therapieresistenter LN. Dennoch werden derzeit weitere Biologika bei LN-Patient*innen getestet, die in Zukunft eingesetzt werden könnten.
